# Male breast cancer: a 30 year retrospective analysis from a tertiary cancer care centre

**DOI:** 10.3332/ecancer.2023.1551

**Published:** 2023-05-18

**Authors:** Abhishek Soni, Yashpal Verma, Ashok Chauhan, Paramjeet Kaur, Vivek Kaushal, Diptajit Paul

**Affiliations:** Department of Radiation Oncology, Pt. B. D. Sharma PGIMS, Rohtak 124001, Haryana, India

**Keywords:** breast cancer, chemotherapy, male, uncommon, radiotherapy

## Abstract

**Background:**

Male breast cancer (MBC) is one of the rare malignancies that account for less than 1% of all malignancies in males. However, the clinicopathological characteristics of MBC are not entirely similar to female breast cancer; but still, it is treated in line with the female breast cancer protocols.

**Aims:**

To retrospectively analyse trends in MBC as to its distribution, presentation, treatment, and outcome.

**Material and method:**

A total of 106 patients with MBC from 1991 to 2020 were analysed retrospectively. Frequency distribution analysis of the demographic and clinicopathological data and treatment variables was done.

**Results:**

Median age of presentation was 57 years; ranging from 30 to 86 years. Either of the sides was almost equally affected with an R: L ratio of 1.2:1. The average duration of complaint was 26.2 months (range 1–240 months). History of gynaecomastia was noted in 18 patients, significant benign prostate hypertrophy in 13, and hypertension needing medical treatment in 14 patients. The majority of the patients were smokers (72/106) and alcoholics (43/106). Five patients reported positive family history. 21 patients had metastatic disease at presentation and received palliative treatment. Stage II was seen in 36.8%, stage III in 43.4%, and stage IV in 19.8% of patients. Node positives were 63.2%. Pathology was invariably (90.5%) infiltrative ductal carcinoma. Radiation was administered in 85.8% of the patients, chemotherapy in 72.6% of patients, and hormonal treatment was given in 47.2% of patients. The median overall survival (OS) was 78 months. OS at 5 and 10 years was 78% and 58% respectively.

**Conclusion:**

Despite the possibility of MBC being apparent at an early stage, patients present with locally advanced disease. Radical surgery with adjuvant/neoadjuvant chemotherapy and adjuvant radiotherapy remains the gold standard. Cancer education campaigns must be run to catch the early disease and to radically treat the disease.

## Introduction

Male breast cancer (MBC) occurs all over the world, but the highest incidence is seen in sub-Saharan countries [1]. MBC is rare cancer representing 1% of all breast malignant neoplasms analysed every year in the United States. In 2021, 2,670 new cases of MBC were analysed in the United States, and around 500 men died from it [2]. The lifetime risk of breast cancer is around 1 out of every 1,000 for a man, while it is around 1 out of 8 for a female. Around the world, the female-to-male incidence ratio is 125:1 [3]. As of now, while the rate of female breast cancer has decreased by 42%, a 28% decline in MBC has been observed [4]. The incidence of breast cancer increases consistently with age in males as well as in females. The typical age of new breast cancer incidence is 5 years more for males (67 years) than for females (62 years) [5]. Other risk factors for MBC incorporate a family background of breast cancer, dark ethnicity, breast or chest exposure to radiation, conveying an inclining germline hereditary change (e.g., BRCA2, BRCA1, CHEK2, PALB2), utilisation of exogenous oestrogen, and diseases related with hyperestrogenism (e.g., Klinefelter syndrome) [3]. Men with Klinefelter condition (XXY) have a 14–50 times risk of developing MBC and account for 3% of all MBC cases [6]. Men with persistent liver issues like cirrhosis, constant liquor abuse, and schistosomiasis; history of mumps, orchitis, undescended testis, or testicular injury; and feminisation, whether hereditary or ecological, are at more risk of developing breast cancer. Gynaecomastia itself does not give an impression of being a single risk factor [6].

There are some lacunae with regard to the ideal management of MBC and its treatment has been extrapolated to a great extent from research led on female breast cancer. Studies that focus on MBC should subsequently recommend the standard of care, but till now they have not been able to address all important questions regarding MBC. There are no randomised studies because of the low number of patients continued in any institution and retrospective studies are additionally not very many. Initial trials proposed that MBC had a poor prognosis in comparison to breast cancer in females [7–9]. The MBC treatment is a combination of surgery, radiation, chemotherapy, and hormonal therapy. The lack of breast tissue in men makes it challenging to achieve safe margins in MBC, especially in small tumors. In India, MBC patients present at a locally advanced stage and it is very difficult to achieve a negative margin. Thus, it mandates adjuvant treatment in MBC patients.

Indications for postmastectomy radiotherapy (PMRT) in MBC are in accordance with the guidelines for female breast cancer. PMRT seems to decrease locoregional recurrence (LRR) in MBC; in any case, the effect on overall survival (OS) is unknown [10–13]. Few studies have depicted the advantage of PMRT in MBC patients [14–17]. Many retrospective studies assessed the role of adjuvant hormonal treatment, and these studies have demonstrated that most MBC patients can benefit from adjuvant tamoxifen in terms of recurrence and survival [18–22]. Although chemotherapy has been indicated to treat high-risk patients with MBC, there is a lack of data to help it. A few studies recommended that adjuvant and systemic treatment in MBC ought to be equivalent to female breast cancer [18–20, 23]. In this review, we retrospectively analysed the clinicopathological characteristics and treatment outcomes of MBC patients at a single institution. The hallmark of the present article is that it demonstrates the MBC data of 30 years’ time period, which, to the best of our knowledge, is the first study on MBC in India spanning over three decades.

## Materials and methods

A total of 8,154 breast cancer cases were registered from 1991 to 2020, out of which 106 MBC cases were identified constituting 1.3% of total breast cancer cases. These were retrospectively analysed for patient‑related characteristics such as age, comorbidity, family history, tumour size, nodal status, stage, histology, oestrogen/progesterone, and human epidermal growth factor receptor 2 (HER‑2) status (Tables 1 and 2). All parameters were entered into a computerised database. As ours is a government institution, immunohistochemistry could not be performed in all patients due to logistic issues and also it was not available in the early phase of the study.

PMRT was delivered to patients with a T3 tumour, close/positive margins, and positive nodes. Standard two tangential opposed fields were used to irradiate the chest wall (CW). Radiotherapy was delivered with 50 Gy in 25 fractions over 5 weeks to CW. The dose was prescribed at the midpoint of the central axis. For supraclavicular fossa, the dose delivered was 50 Gy in 25 fractions over 5 weeks with a single‑incident field. Radiation therapy was delivered using a Cobalt-60 machine. Tamoxifen was prescribed to estrogen receptor (ER)/progesterone receptor (PR)‑positive patients for 5 years as per female breast cancer guidelines and letrozole which was prescribed for a further 3–5 years in females, was not prescribed in MBC cases [5]. Patients were followed up at regular intervals: every 3 months till 1 year, every 4 months till 3 years, every 6 months till 5 years, and yearly thereafter. Further tests were done only if they had symptoms or evidence of recurrent or metastatic disease. Treatment outcomes analysed were LRR, disease‑free survival (DFS), and OS. LRR was defined as any recurrence in the skin or soft tissue over CW or a recurrence in the regional lymphatic sites (axilla, internal mammary nodes, infraclavicular, and supraclavicular). DFS was defined as the time duration from surgery to the first recurrence. OS was defined as the time duration from pathologic diagnosis to death or last follow‑up with any death defined as an event. DFS and OS were estimated using Kaplan–Meier method and compared between patients receiving and not receiving adjuvant treatment using the log‑rank test. All statistical tests were two-tailed, and the differences were considered statistically significant if *p* < 0.05. Statistical analysis was performed using Statistical Package for Social Sciences software’s recent version.

## Results

Table 1 shows the patients characteristics. The median age was 57 years. Most (67%) of the patients belonged to a rural region. In all patients, the lump was the presenting complaint and the other complaints were pain and discharge. More than half of the patients presented with right side MBC. The mean duration of symptoms was 26.2 months. Comorbidities and family history were present in 42.5% and 4.7% patients, respectively. Out of 106 patients, 67.9% were chronic smokers and 40.6% were alcoholics. Table 2 depicts the tumour characteristics of the patients. The mean tumour size was 5.8 cm. The nipple was involved in 57.5% patients. The majority (90.5%) patients had invasive ductal carcinoma histology, others were undifferentiated and papillary. Out of 106 patients, the most common stage was T3 (51.9%), and the least common was T4A (7.5%). A total of 63.2% of patients were node positive. Out of 67 node-positive patients, 62.7% were N2A, 25.3% were N3B and 12% were N3C. 21 patients (19.8%) presented with metastasis. Out of 106 patients, 30.2% presented in stage IIIA, 24.5% in stage IIA, 19.8% in stage IV, 12.3% in stage IIB, 11.3% in stage IIIC, and 1.9% in stage IIIB. Early, locally advanced, and metastatic diseases were seen in 36.8%, 43.6%, and 19.8% patients, respectively. ER, PR, and HER-2neu positive patients were seen in 41.5%, 25.5%, and 8.5% patients, respectively. ER, PR, and HER-2neu cannot be performed for all patients due to logistic issues.

**Figure 1.**
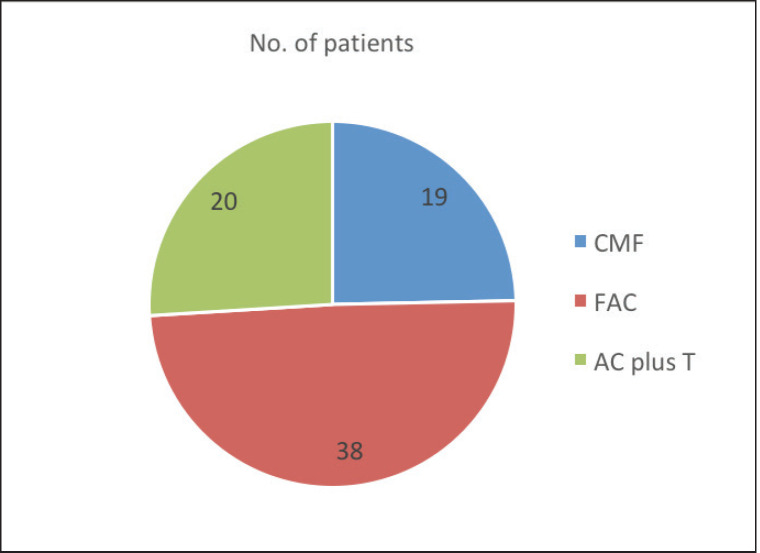
Chemotherapy regimens administered to the MBC patients.

Adjuvant radiotherapy, chemotherapy, and tamoxifen were received by 85.8%, 72.6%, and 47.2% men, respectively. Chemotherapy regimens used were CMF (Cyclophosphamide 600 mg/m^2^, Methotrexate 40 mg/m^2^, and 5‑FU 600 mg/m^2^) in 24.7% patients in the early part of the study period till 1996, FAC (5‑FU 600 mg/m^2^, Adriamycin 50 mg/m^2^, and Cyclophosphamide 600 mg/m^2^) in 49.4% patients till 2008, and anthracyclines and taxanes in 25.9% patients from 2008 afterwards (Figure 1). The median number of courses of chemotherapy was 4.5. Among 21 patients with metastatic disease, 16 men received chemotherapy, 4 became operable, 8 also received radiotherapy and tamoxifen and 5 patients with poor general condition were given only supportive care. In radically treated 85 men, neoadjuvant chemotherapy with FAC and FAC + taxanes regimen was given to 49.4% and 25.9% of patients, respectively. Complete response and PR were seen in 65.9% and 34.1% of patients, respectively. Mastectomy was done in 85.9% and wide local excision in 14.1% of patients.

**Figure 2.**
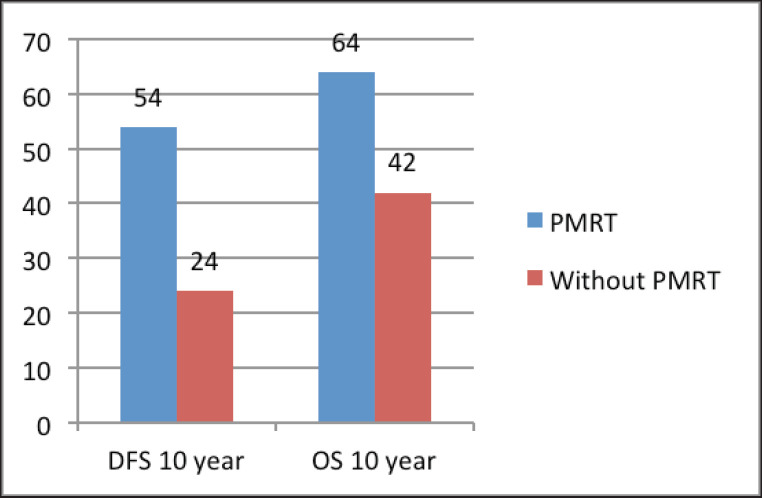
DFS and OS at 10 years with and without PMRT.

**Figure 3.**
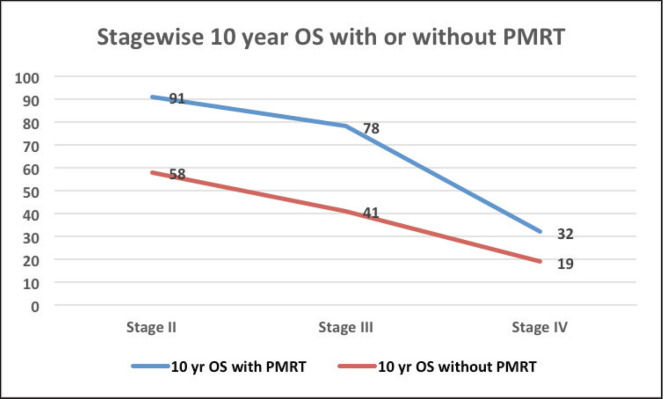
OS at 10 years with and without PMRT in various stages of MBC.

**Figure 4.**
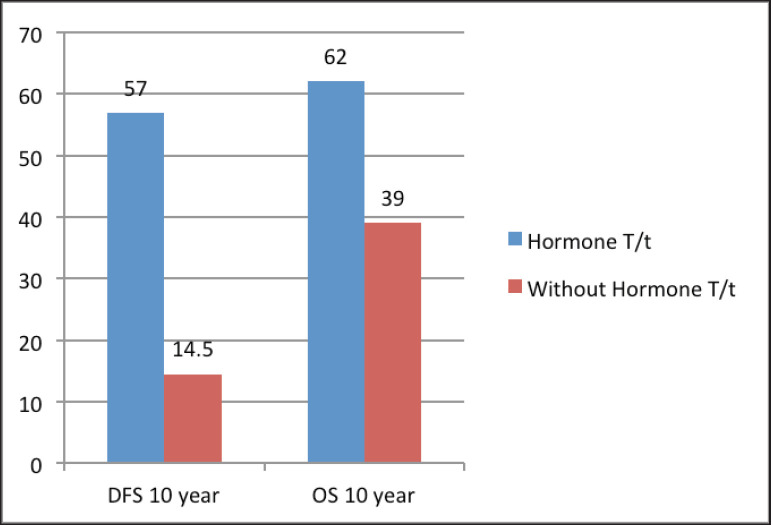
DFS and OS at 10 years with and without hormonal treatment.

The median follow‑up was 60 months (range of 4–278 months). For the entire cohort, LRR occurred in 9.4% and distant metastasis in 19.8% of patients. PMRT reduced LRR, and improved OS and DFS in low‑ as well as high‑risk patients. For the entire cohort, the median DFS was 43 months, and the 5 and 10‑year survival rates were 78% and 58%, respectively. The median OS was 78 months. DFS at 10 years was 54% and 24% with and without PMRT (*p* = 0.007), respectively (Figure 2). OS at 10 years was significantly better in men with PMRT (64%) as compared to 42% without PMRT (*p* = 0.022) (Figure 2). The OS was better in the early stages and decreased in the advanced stages (Figure 3). Statistically significant improvement in OS with PMRT was seen across all stages and 10-year OS for stage II was 91% versus 58%, for stage III – 78% versus 41% and for stage IV – 32% versus 19%, with and without PMRT, respectively, *p* < 0.05 (significant). 10-year DFS for stage II was 72% versus 41%, for stage III – 55% versus 26% and for stage IV – 19% versus 11%, with and without PMRT, respectively, *p* < 0.05 (significant). Hormonal therapy also significantly improved DFS and OS at 10 years (57% versus 14.5%, *p* = 0.004 and 62% versus 39%, *p* = 0.045, respectively) (Figure 4). Only 1 (1%) patient developed a second malignancy in the contralateral breast after 8 years of follow‑up.

## Discussion

The incidence of MBC patients in the present study was 1.3%, which was slightly higher than the worldwide average of 1%, but it is in close relation to the Indian study from Patna, Bihar by Pawar *et al* [24]. However, higher incidence is reported by Shah *et al* [25] (4.1% from Kashmir) and Khandelwal *et al* [26] (1.9% from Punjab). This might be due to geographical variations. In the present study, the patients were 10 years younger than those demonstrated in the Western studies. The median age in this study was 57 years [7] versus 67 years from West literature [25]. In the present study, patients presented late with mean duration of symptoms 26.2 months and in the advanced stage with mean tumour size of 5.8 cm contrasted with Western patients [25]. The patients in this study were also included from the 1990s and at that time, the Indian male patients were too shy to report to the physician regarding any changes in the breast; and so, the mean duration of symptoms was so high. Most patients presented at a locally advanced stage because of ignorance, obliviousness, elective medicines use (desi and ayurvedic treatment), and distant cancer centres. A family history was seen in 4.7% of patients in this study. Nigerian study by Ahmed *et al* [28] reported 2.5% familial risk. The familial risk from Indian studies was 15.4% from Bengaluru [28], 10% from Delhi by Gogia *et al* [30], and no familial risk was reported by Sundriyal *et al* [34] from Delhi. This might be because of topographical contrasts. There is not a lot of information from India particularly on familial cancer risk in MBC as Indian patients might have less familial risk. Another explanation might be underreporting, yet its possibilities are less because there is a standard format for recording the patient history and it incorporates family history as one of its parts. Although recommended for such cases, still genetic testing for BRCA mutation was not possible in the present study due to logistic issues.

In this study, approximately three fourth of the total MBC patients had central quadrant disease, and 57.5% had nipple involvement. These findings are similar to the available literature [31]. More than 90% of patients were having infiltrative ductal carcinoma (IDC) histology, which is consistent with female breast cancer and study by Khandelwal *et al* [26] at Tata Medical Centre. The literature reports that 90% of MBCs express ER and 81% express PR, contrasted with 75% and 66% in females with BC [23, 25]. Some Indian studies also reported ER/PR positivity in the range of 77%–89% [28–30, 32, 33]. However, the present study reported a relatively less rate of ER/PR expression. The reason behind this was no hormone receptor testing facilities in our institution in the early 1990s. Incidence of triple-negative MBC patients was 20.8% in the present study which was consistent with 22% incidence in female breast cancer [29].

In our institution, PMRT is prescribed to patients when indicated like in T3/T4 tumour, close/positive margins, and positive nodes [32]. In this study, PMRT was administered to most MBC patients because of the locally advanced stage at initial presentation. PMRT was seen to improve DFS and OS. LRR rates without PMRT was 14%–25% in low-risk patients and 25%–50% in high-risk patients. The LRR rate with PMRT was 3.5%–13%. Yu *et al.* [13] from Canada reported improved LRR with PMRT in patients with high-risk MBC patients (positive node, high stage, ≤2 mm or positive margin). But it did not demonstrate OS benefit with PMRT. LRR rate without PMRT in the present study was higher (32.7% without PMRT and 9.4% with PMRT) in comparison to some other studies [9, 17, 34, 35]. This might be due to the bigger tumour size and advanced stage of presentation in our patients. The LRR reported to be 3.5% by Chakravarthy and Kim [9], 4.7% with PMRT and 11.5% without PMRT by Cutuli *et al* [17], 6.9%–10.3% in low-risk patients without PMRT & 17%–20% in high-risk patients by Yu *et al* [13], 11.3% with PMRT by Selcukbiricik *et al* [37] from Turkey, 18% with PMRT by Perkins *et al* [16]. Cutuli *et al* [36] reported LRR rates of 7.3% with PMRT and 13% without PMRT. Conversely, Hultborn *et al* [38] reported an LRR rate of 30% in patients with greater than equal to 5 cm tumour or metastases with perinodal development. Iyer *et al* [39] demonstrated that neoadjuvant CTRT in MBC in limited patients reduced LRR and improved OS and DFS. The 5-year local recurrence-free survival (LRFS) in this study was 63%, which is similar to those demonstrated in the literature (55%–69%) [10, 12–17, 21, 34, 39–43]. The LRFS rate was better in patients treated with PMRT in comparison to surgery alone (*p* = 0.038), but there was no survival difference (*p* = 0.72) [14]. Our outcomes are in line with different studies of MBC patients and in favour of PMRT versus surgery alone [13, 17, 34, 41, 42].

Neoadjuvant chemotherapy showed no advantage in DFS or OS. DFS at 10 years with and without neoadjuvant chemotherapy was 62% and 57%, respectively (*p* = 0.66). Additionally, OS at 10 years with and without neoadjuvant chemotherapy was 64% and 59%, respectively (*p* = 0.77). A total of 77 (72.6%) patients went through chemotherapy in this study. Recurrence rates were lower in patients who got both chemotherapy and hormonal treatment contrasted to chemotherapy alone 35% versus 78%. Giordano *et al* [23] reported advantage of chemotherapy with 10-year OS of 43% in patients with positive nodes.

Standard hormonal therapy in ER/PR-positive patients is tamoxifen. A few studies have shown a decreased risk of local recurrence and mortality in MBC patients who got tamoxifen [35, 44, 45]. Ribeiro and Swindell [45] reported that 5-year DFS and OS were 61% and 41% versus 56% and 28% with 1–2 years tamoxifen post mastectomy versus mastectomy alone, respectively. Similarly, Goss *et al* [46] reported advantage in both DFS and OS. In the present study, tamoxifen was prescribed for 5 years and hormonal therapy improved 10-year DFS (57% versus 14.5%) and 10-year OS (62% versus 39%); which is in similar lines to the literature [23, 25, 44, 45]. This might be due to the fact that hormone receptor-positive MBC patients have better prognosis as compared to other subtypes. There is no data on the extended utilisation of tamoxifen in MBC. If tamoxifen is contraindicated, aromatase inhibitors are not administered as sole agent, however, aromatase inhibitors may be co-administered with gonadotropin-releasing hormone analogues [5].

The limitation of the present study was retrospective nature with a small number of patients from a single institution. In one-third of our patients, ER/PR status was not known, yet the larger part (70%) were getting hormonal treatment. Of four different studies with a limited number of patients and follow-ups from India, three announced that ER/PR was communicated in 77%–89% of MBC patients [28–30, 46]. Subsequently, the administration of tamoxifen to 70% of patients in this study might be legitimate. In any case, three different studies did not report the result of PMRT or other adjuvant treatments [28, 30, 46]. Due to retrospective nature and small sample size, it is difficult to analyse the effects of different adjuvant treatment strategies on LRR and OS rates. Furthermore, the present study was conducted over three decades and thus, change in demography and culture affected patient’s characteristics. Also, the evolving diagnostic approaches with ER/PR/HER-2-neu status and imaging modalities, staging system, and treatment strategies affected results of the present study.

The strength of our study is that it is the largest data set from India on MBC with a good median follow‑up of 60 months in all patients treated on a cobalt machine with conventionally fractionated radiotherapy. The recurrence rate in MBC patients in this study is equivalent to female BC patients [47, 48]. The available literature also supports that patient gender does not seem to be a prognostic factor in local disease recurrence and PMRT further improved DFS and OS in female BC; therefore, it is reasonable to consider PMRT in MBC patients with high‑risk features [32, 33, 48–56].

## Conclusion

MBC patients present at a locally advanced stage with a long median duration of symptoms despite the possibility of being diagnosed at an early stage. Radical surgery with chemotherapy and post-mastectomy radiation treatment remains the standard practice as in the females. Patients in this study were younger as compared to the Western series. PMRT reduced the LRR rate in MBC. PMRT and hormonal therapy significantly improved DFS and OS in MBC patients. However, ER/PR-positive patients might have been responded better to hormonal treatment. Chemotherapy had no impact on DFS and OS. However, future prospective trials with a large sample size may enlighten the path to successful treatment with minimal recurrence. This study necessitates health education and cancer awareness in the rural parts of India as 70% of the population of India belongs to a rural background. So, effective cancer education campaigns and/or programs must be run for early presentation and better cure of MBC.

## Conflicts of interest

Nil.

## Funding

Nil.

## Figures and Tables

**Table 1.** Patient characteristics of the MBC patients.

Characteristic		No. of patients (%)
Total number of patients		106
Median age (range)		57 (30–86) years
Rural distribution		71 (67%)
Laterality	Right Left Bilateral	56 (52.8%) 48 (45.3%) 02 (1.9%)
Duration of symptoms – Mean (range)		26.2 (1–240) months
Comorbidities		45 (42.5%)
	Gynecomastia	18 (17%)
	BPH	13 (12.3%)
	HTN	14 (13.2%)
Family history		05 (4.7%)
Chronic smokers		72 (67.9%)
Alcoholic		43 (40.6%)

**Table 2.** Tumour characteristics of the MBC patients.

Characteristic		No. of patients (%)
Tumor size – Mean (range)		5.8 (2–10) cm
Nipple involvement		61 (57.5%)
Histology	IDC Undifferentiated Papillary	96 (90.5%) 06 (5.7%) 04 (3.8%)
Tumor	T2	28 (26.4%)
	T3	55 (51.9%)
	T4a	08 (07.5%)
	T4b	15 (14.2%)
Node	N2a	42 (62.7%)
	N3b	17 (25.3%)
	N3c	08 (12%)
Stage	II	39 (36.8%)
	IIa	26 (24.5%)
	IIb	13 (12.3%)
	III	46 (43.4%)
	IIIa	32 (30.2%)
	IIIb	02 (1.9%)
	IIIc	12 (11.3%)
	IV	21 (19.8%)
ER		44 (41.5%)
PR		27 (25.5%)
Her-2-neu	Positive	09 (8.5%)
	Negative	46 (43.4%)
	Unknown	51 (48.1%)
TNBC		22 (20.8%)
Early disease		39 (36.8%)
Locally advanced disease		46 (43.6%)
Metastatic disease		21 (19.8%)
